# Mechanical and Thermal Properties of Dental Composites Cured with CAD/CAM Assisted Solid-State Laser

**DOI:** 10.3390/ma11040504

**Published:** 2018-03-27

**Authors:** Roberto De Santis, Antonio Gloria, Saverio Maietta, Massimo Martorelli, Alessandro De Luca, Gianrico Spagnuolo, Francesco Riccitiello, Sandro Rengo

**Affiliations:** 1 Institute of Polymers, Composites and Biomaterials—National Research Council of Italy, V.le J.F. Kennedy 54—Mostra d’Oltremare Pad. 20, 80125 Naples, Italy; rosantis@unina.it; 2 Department of Industrial Engineering, Fraunhofer JL IDEAS—University of Naples Federico II, P.le Tecchio 80, 80125 Naples, Italy; smaietta@unina.it (S.M.); massimo.martorelli@unina.it (M.M.); 3 Department of Industrial and Information Engineering, University of Campania “Luigi Vanvitelli”, via Roma 29, 81031 Aversa, Italy; alessandro.deluca@unicampania.it; 4 Department of Neurosciences, Reproductive and Odontostomatological Sciences, University of Naples Federico II, Via Pansini, 5, 80125 Naples, Italy; gianrico.spagnuolo@gmail.com (G.S.); riccitie@unina.it (F.R.); sandro.rengo@unina.it (S.R.)

**Keywords:** computer-aided design/computer-aided systems, dental materials, composites, mechanical properties, thermal properties, laser

## Abstract

Over the last three decades, it has been frequently reported that the properties of dental restorative composites cured with argon laser are similar or superior to those achieved with conventional halogen and light emitting diode (LED) curing units. Whereas laser curing is not dependent on the distance between the curing unit and the material, such distance represents a drawback for conventional curing units. However, a widespread clinical application of this kind of laser remains difficult due to cost, heavy weight, and bulky size. Recently, with regard to the radiation in the blue region of the spectrum, powerful solid-state lasers have been commercialized. In the current research, CAD (computer-aided design)/CAM (computer-aided manufacturing) assisted solid-state lasers were employed for curing of different dental restorative composites consisting of micro- and nanoparticle-reinforced materials based on acrylic resins. Commercial LED curing units were used as a control. Temperature rise during the photopolymerisation process and bending properties were measured. By providing similar light energy dose, no significant difference in temperature rise was observed when the two light sources provided similar intensity. In addition, after 7 days since curing, bending properties of composites cured with laser and LED were similar. The results suggested that this kind of laser would be suitable for curing dental composites, and the curing process does not suffer from the tip-to-tooth distance.

## 1. Introduction

Light activated composites [1] represent the most popular choice in dental restoration practice [2] because these materials, used in conjunction with a light curing unit (LCU) [3], allow for the on-demand process of polymerisation. This approach offers the clinical advantage of extended working time, thus promoting precise material placement. The dental restorative composites consist of an organic matrix and an inorganic reinforcement. The most common polymeric matrix is mainly based on bisphenol A glycidyl methacrylate (Bis-GMA) or ethoxylated bisphenol A dimethacrylate (EBPADMA) and diluent monomers, such as triethylene glycol dimethacrylate (TEGDMA), 1,4-butanediol dimethacrylate (BDDMA), and urethane dimethacrylate (UDMA) [4,5,6,7]. Polymerisation is induced by a photoinitiator system and its interaction with light. Camphorquinone (CQ), in conjunction with a tertiary amine, represents the most popular photoinitiator system that can be activated by a blue light [8,9,10].

The light curing process is an important factor affecting the performance of the composite restoration; in particular, mechanical properties strongly depend on the depth of cure and degree of conversion [11,12,13]. During the last decades, a variety of light curing units (LCUs) have been developed in order to improve the quality of restorative materials [3,13]. LCUs are generally characterised by an emission bandwidth mainly centred at about 470 nm, which is the maximum absorption wavelength for CQ. Quartz–tungsten–halogen unit has been extensively used to cure dental composites. More recently, light emitting diode (LED) represents an alternative light source to traditional halogen units [3]. The wavelength spectrum of LED based LCUs is narrow, and precisely calibrated on CQ absorption wavelength, while QTH based LCUs emit light over a wide spectral range (400 nm–700 nm) [14]. As a consequence, LED units have a low electric power consumption to achieve radiation [13].

Dental light sources based on light amplification by stimulated emission of radiation (laser) have also been developed. Basically, argon laser based LCUs have been adopted to photo-cure dental composites, and a wide literature has been developed over the past three decades [3,15,16,17,18,19,20,21]. The main feature of this LCU is a beam of monochromatic and coherent light, and for the argon laser, the very narrow wavelength spectrum with a peak at about 488 nm [16]. The light intensity level of laser LCU is much higher than that of other type of LCUs. As a consequence of these properties, it has been reported that laser curing may improve the performance of dental composites for specific clinical requirements [17]; nevertheless, laser LCUs are expensive when compared with other type of LCUs, such as argon ones [17]. However, solid-state lasers emitting in the blue spectrum have been recently developed. Diode-pumped solid-state lasers benefit from the same technology, which has prompted the LED light source to the clinical practice. These lasers are based on GaN and InGaN, and like the LED units, they are inexpensive, with low electric power consumption [18].

As reported in the literature, results concerning mechanical properties of dental composites cured with halogen, LED, or laser units are conflicting. Properties of composites cured using argon laser line at 488 nm with 250 mW power and 10 s exposure time are better than those obtained with conventional LCUs [19,20], while properties of composites cured with higher power laser diode (675 mW) have been found to be not different from those recorded for LED polymerisation [21].

Conflicting results were also related to the depth of cure achieved with halogen, LED, or laser units. Similar penetration depth, investigated up to 2 mm, were recorded for argon laser with 25 mW power and halogen units [22]. Instead, in comparison with halogen light, a better depth of cure has been observed for argon lasers as the intensity of the two light sources was set at 20 mW/cm^2^ [23]. For composite thickness higher than 2 mm, the depth of cure obtained with argon laser at 250 mW has been found similar to that of halogen light [24].

Another important feature of laser curing units is related to temperature rise in the composite during the polymerisation process. In the pulp cavity, temperature increase is of paramount importance, and thermal injury thresholds represent the main limit to the power level which can be safely delivered [25,26,27]. Temperature rise occurring during composite photopolymerisation with argon laser [23] and diode laser [18] is lower than that of conventional LCUs. Moreover, pulsed laser curing [28] should further decrease the temperature rise and the curing time.

A variety of theoretical analyses and experimental tests have been carried out in the past half century, in order to predict or to determine mechanical properties of dental composites [3,22,24,29,30,31]. As stiffness and strength are concerned, classical mechanical analyses, such as bending tests, represent the most popular approach to directly measure the elastic modulus and the maximum stress [14,15,20]. The preparation of specimens suitable for bending tests is tricky, as they are bar-shaped with a length which is higher than the diameter of the fibre tip of LCUs. For this reason, using conventional or laser LCUs, bending specimens are cured through many “polymerisation steps” [15]. A minimum of two polymerisation steps are required to cure the whole bending specimen with a conventional LCU [14], and up to five overlapping sequences have been necessary when using the laser [15,20]. This clearly causes the lack of homogeneity and the presence of several interfaces, thus leading to a process which is difficult to reproduce and standardise, especially if laser sources are concerned.

While a huge literature has been developed on photopolymerisation with argon lasers, little is known on the use of solid-state lasers [18,20,21,24]. Literature concerning argon laser suggests a lower temperature rise during photopolymerisation, however, the effect of solid-state lasers is still missing. Accordingly, the effect of solid-state lasers on temperature rise and mechanical properties was analysed in the current research. Furthermore, we also adopted a novel approach as a standardised method for curing of dental restorative composites using CAD (computer-aided design)/CAM (computer-aided manufacturing) systems [32,33,34] equipped with LCUs.

## 2. Materials and Methods

### 2.1. Dental Composites

Four kinds of dental restorative composites, consisting of micro- and nanoparticle-reinforced materials based on acrylic resins and CQ as a photoinitiator, were used. The specifics of the analysed materials are reported in Table 1 [26,29,35].

### 2.2. Light Curing Units

Table 2 lists the light curing units (LCUs) employed for light curing materials.

### 2.3. Optical Measurements

Wavelength distribution of the LCUs was measured using a compact spectrometer (350–700 nm, CCS100/M, ThorLabs, Newton, NJ, USA) equipped with FT030 fibre (reinforced Ø3 mm Furcation Tubing, Newton, NJ, USA), CCSAC cosine corrector and ThorLabs OSA software. Optical power of the LCUs were measured using a radiometer (LED, Demetron, Kerr, CA, USA), a compact power, and energy meter console (PM100D, ThorLabs) equipped with a sensor (S121C, ThorLabs), and connected to the PMD100D software running under LabView.

In order to map the spatial light intensity of the laser curing unit, the optical fibre of the spectrometer was fixed to a 3-axis CAD/CAM system moved by stepper motor with resolution of 1 µm [33], and mapping was obtained at a pitch of 152 µm. The procedure adopted for mapping the laser beam profile is similar to the pinhole technique [36,37]. Even though this approach is seen as time-consuming [36], it is also considered as a versatile technique to profile beams with complex distribution [38], such as in the case of solid-state lasers [39]. With regard to the values of light intensity measured for the employed solid-state laser, data collected through the spectrometer in form of arbitrary intensity distribution [36,38,40] were converted to mW/mm^2^ according to the following equation:

(1) $$P=\sum_{x,y}I_{x,y}\cdot A_{x,y}=\sum_{x,y}I_{M}C_{x,y}\cdot A_{x,y}$$

where *P* is the output optical power measured by the power meter, *I_x,y_* is the light intensity, *A_x,y_* is the unit area used to map light intensity (152 × 192 µm^2^), *I_M_* is the intensity as variable to be determined, and *C_x,y_* is the arbitrary intensity distribution measured by the spectrometer. In the Equation (1), the arbitrary intensity distribution *C_x,y_* represents the ratio between *I_x,y_* and *I_M_*, and *I_M_* is the maximum intensity at the centre of the beam spot [36,41].

The power output of a variety of light sources was measured with a calibrated instrument (a radiometer—LED Demetron–Kerr, a compact power and energy meter console—PM100D, ThorLabs). Generally, the calibration is performed using a signal with known power or measured by a power meter [42].

### 2.4. Specimen Preparation

Teflon moulds with a length of 40 mm, width of 1 mm, and depth of 1 mm were employed to manufacture the composite specimens (27 × 1 × 1 mm^3^). A transparent Mylar strip was used on the top of the specimen, and 2.5 kg (dead weight) was applied for 60 s before light curing. Two curing modes (Mode I and Mode II) with different light energy doses were investigated.

**Figure 1:**
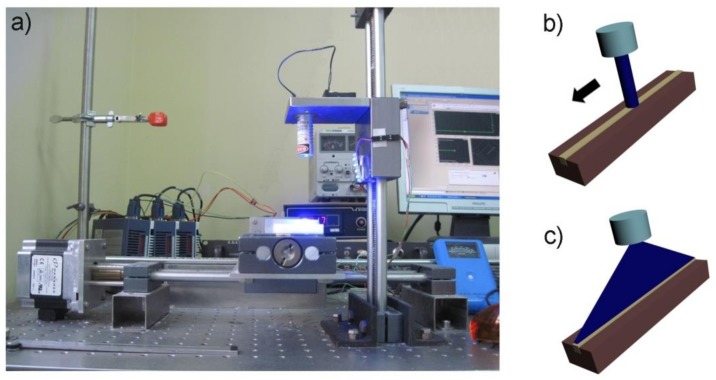
Set-up adopted for curing composite specimens. (**a**) CAD (computer-aided design)/CAM (computer-aided manufacturing) system used for positioning of the light beam of the LCUs; (**b**) Mode I curing. The relative movement at a constant speed between the LCU and the Teflon mould containing the composite material allowed to reproduce and standardise the photopolymerisation process; (**c**) Mode II curing. The laser and a cylindrical line generating lens were used to produce a line-shaped beam.

With regard to the first curing mode (Mode I), photopolymerisation of each composite material was carried out by using the 3-axis CAD/CAM system driven by LabView software (Figure 1).

The laser or the LED Mectron unit was placed on the arm of the apparatus (*z*-axis), while the Teflon mould was fixed on the *x*,*y* platform. The relative movement at a constant speed between the LCU and the Teflon mould allowed for reproduction and standardisation of the photopolymerisation process. The optical energy involved for the curing process was computed according to the following equation:

(2) $$E=\int_{0}^{t_{f}}I\cdot w\cdot x(t)\;dt\;with\;\{\begin{array}{lll} x(t)=v\cdot t & for & 0<t<t_{1}\\ x(t)=x & for & t_{1}<t<t_{2}\\ x(t)=x-v\cdot t & for & t_{2}<t<t_{f} \end{array}$$

where *I* is the light intensity, *w* is the specimen width, *x*(*t*) is the effective light beam thickness illuminating the specimen, *x* is the laser beam width or the diameter of the blue LED fibre curing units, *v* is the relative speed, *t*_1_ = *x*/*v*, *t*_2_ = (*L_T_* − *x*)/*v*, *L_T_* and *t_f_* are the total length of the specimen and the total exposure time, respectively. In particular, *t*_1_ represents the time required by the light source to completely engage the specimen (attack phase), while *t*_2_ is the time required to completely disengage the specimen (release phase). The speed *v* was set in order to provide an optical energy of 4.85 J and 4.89 J for the laser and the blue LED curing units, respectively.

**Figure 2:**
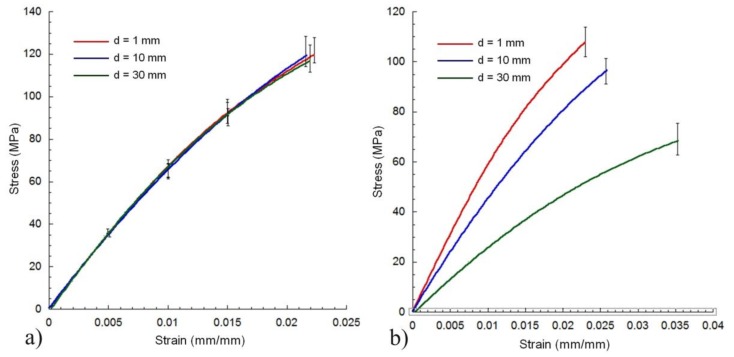
Effect of the distance between the LCU and the restorative composite (EPH) on the bending behaviour/stress–strain curves for laser cured (**a**) and LED cured (**b**) composites. The distance was set at 1, 10, and 30 mm.

In order to verify the independence of the distance between the laser diode and the composite, preliminary tests on the restorative composite (EPH) were performed, and the distance was set at 1, 10, and 30 mm (Figure 2). In terms of maximum stress or elastic modulus, no statistically significant differences were found using different values of the distance between the laser and the restorative composites (ANOVA followed by Tukey’s test at a critical value of 0.05). Based on these preliminary results, Mode I laser curing was performed using an arbitrary distance of 30 mm.

Concerning the second curing mode (Mode II), the laser and the LED Light Engine units were used. The laser and a cylindrical line generating lens with a diameter of 8 mm and thickness of 2 mm were used. This lens produces a line-shaped beam having a Gaussian distribution of light intensity [43,44]. The energy delivered to the specimen was given by the following equation:

(3) $$E=\sum_{x,y}I_{x,y}\cdot A_{x,y}\cdot t_{f}$$

where *I_x,y_* is the intensity at the given *x*,*y* position, and determined according to Equation (1); *A_x,y_* is the unit area; *t_f_* is the exposure time. Thus, the energy provided by the laser unit was 4.93 J.

Instead, with regard to the LED Light Engine unit, a distance of 11 mm between the LCU and the Teflon mould was used. At this distance, the intensity is 450 mW/cm^2^ over the whole length of the specimen (27 mm). Photopolymerisation was carried out for 40 s, and an energy dose of 4.86 J was used.

### 2.5. Temperature Measurements

In order to evaluate temperature profiles in the restorative composites during the curing process, transparent glass moulds and the Fluke Ti10 thermal imaging camera (Fluke, Everett, WA, USA) were used. Figure 1 shows the set-up adopted for curing composite specimens. Three replicates for each composite were cured in glass moulds, and Fluke SmartView software (version 3.0, Fluke, Everett, WA, USA) was used to record temperature profiles.

Maximum values of temperature were analysed, and means were compared by Tukey’s test.

### 2.6. Mechanical Tests

To evaluate the mechanical properties, three-point bending tests were performed at 1 mm/min using a support span of 20 mm. An Instron 5566 testing machine (Instron Ltd., High Wycombe, England) was employed. Five replicates for each composite and for each curing mode were used.

Data were analysed using two-way ANOVA followed by Tukey’s test at a critical value of 0.05.

## 3. Results

### 3.1. Characteristics of LCUs

**Figure 3:**
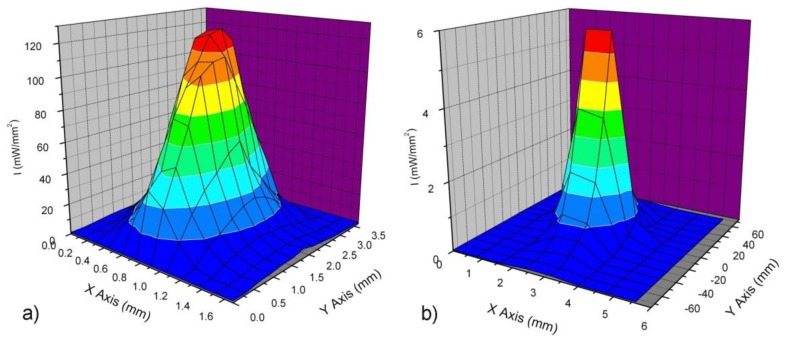
Intensity distribution of the laser beam for Mode I (**a**) and Mode II (**b**) curing.

Figure 3 shows the intensity distribution of the laser beam for Mode I (a) and Mode II (b) curing.

Intensity mapping was performed at a pitch of 152 × 192 µm^2^ and 785 × 1000 µm^2^ for Mode I and Mode II photopolymerisation, respectively. It was found that in the case of Mode I, the dot-shaped beam had a maximum intensity in the centre (Figure 3a), whereas for Mode II, the line-shaped beam showed an almost Gaussian distribution for the light intensity. In addition, it is reported that the minimum energy required for the optimum conversion of light activated resin systems is about 1 J/cm^2^ [45]. According to the optical energy used for photocuring (Table 2), it can be assumed that the energy provided by the LCUs (18 J/cm^2^) was sufficiently high to ensure adequate polymerisation and mechanical properties [45,46,47,48].

### 3.2. Temperature Measurements

Typical temperature profiles in the material cured using different modes and light sources were analysed and reported in the following Figures, where the photograms were taken every 4 s. 

**Figure 4:**
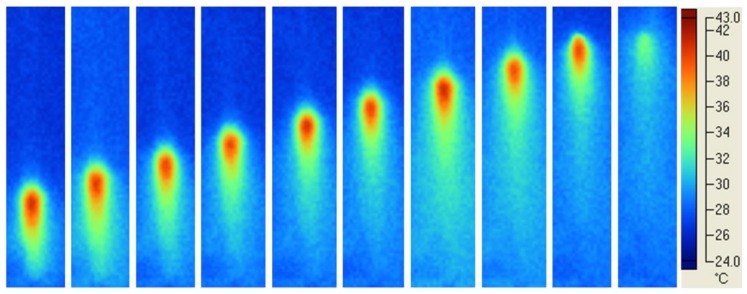
Light curing of EPH composite: typical temperature profiles using Mode I and laser beam. The dot-shaped beam light along the whole length of the specimen was observed. Photograms were taken every 4 s.

Figure 4 shows temperature profiles in the material cured using Mode I and laser beam. At a constant speed, the dot-shaped beam light along the whole length of the specimen was observed during the polymerisation process. 

**Figure 5:**
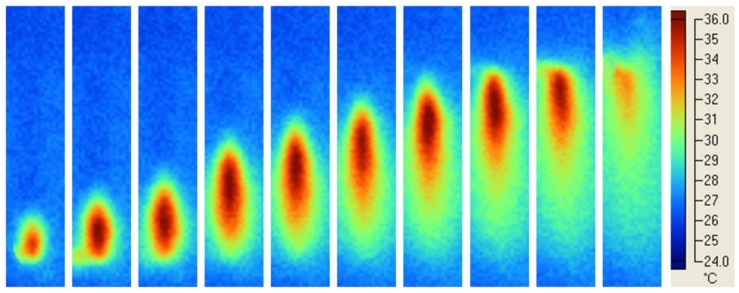
Light curing of DEI composite: typical temperature profiles using Mode I and LED blue Mectron unit. The large spot beam light along the whole length of the specimen was observed. Photograms were taken every 4 s.

In the case of Mode I curing and LED blue Mectron unit, typical temperature profiles in the material are reported in Figure 5. At a constant speed, a large circular beam light (diameter of 8 mm) along the whole length of the specimen was observed during the polymerisation process.

Temperature profiles obtained using the Mode I (Figure 4 and Figure 5) suggested that a soft energy start and a more marked soft energy release were automatically included in the process.

**Figure 6:**
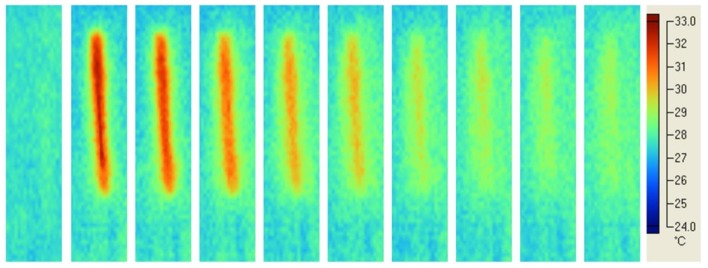
Light curing of CLM composite: typical temperature profiles using Mode II and laser line-shaped beam. The almost uniform distribution of temperature along the whole length of the specimen suggested a uniform polymerisation. Photograms were taken every 4 s.

Typical temperature profiles in the composite material cured using Mode II and laser line-shaped beam are shown in Figure 6. Although the Gaussian light intensity distribution (Figure 3b) was evident, the almost uniform temperature profile along the whole length of the specimen (Figure 6) suggested a uniform polymerisation. Moreover, during the polymerisation process, the photograms suggested that the peak temperature was reached about 8 s after the light curing process began. 

**Figure 7:**
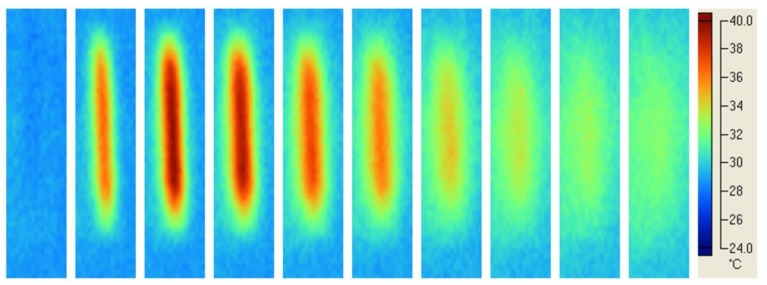
Light curing of SDR composite: typical temperature profiles using Mode II and blue LED Enfis curing unit. The almost uniform distribution of temperature along the whole specimen length suggested a uniform polymerisation of the specimen. Photograms were taken every 4 s.

Figure 7 shows the results in terms of temperature profiles obtained using Mode II and blue LED Enfis curing unit.

The almost uniform distribution of temperature along the whole specimen length (Figure 7) suggested a uniform polymerisation. Compared with the Mode II curing and laser line-shaped beam (Figure 6), a marked ring around the specimen was detected at higher temperature levels for the Mode II curing and blue LED Enfis unit (Figure 7). These higher temperature levels may be due to the higher irradiating area produced by the LED unit, which heated the surrounding mould.

Taking into account the results from Figure 6, the peak temperature was observed at 8 s in the case of Mode II laser curing, whereas qualitatively similar results were found for Mode II LED curing, where the peak temperature was observed later for the SDR composite (Figure 7). Such effect should be ascribed to the different kinetics of polymerisation as a consequence of the different material composition.

Table 3 reports the temperature rise recorded during the photopolymerisation process for the analysed materials, according to the light source and curing mode.

### 3.3. Mechanical Tests

**Figure 8:**
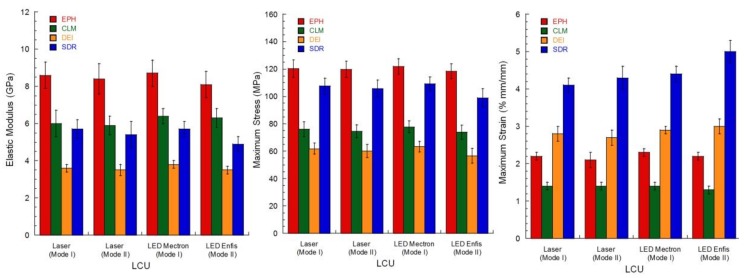
Mechanical properties of restorative composites after 7 days since curing, according to light source and curing mode. The results are reported in terms of mean values, the bar represents the standard deviation.

Table 4 and Figure 8 summarise the mechanical properties evaluated after 7 days since curing with laser and LEDs units according to the Mode I and Mode II polymerisation process.

For a given material, no statistically significant difference was observed if the composite was cured with laser and LED units, also suggesting that, independently from the light source and the curing mode, similar mechanical properties were obtained when similar energy doses were used. 

Furthermore, concerning a specific mechanical property, significant differences were observed among the different restorative composites. Specifically, the EPH composite showed a significantly higher elastic modulus (*p* < 0.05) in comparison with the DEI, CLM, and SDR composites. The bending strength (Table 4) of EPH was also significantly higher (*p* < 0.05) than those of DEI and CLM, whereas no difference was detected between the maximum stress of EPH and SDR cured with the blue laser. Even though the lowest values of modulus and maximum stress were obtained for the DEI composite (*p* < 0.05), the highest values of strain at break were achieved for the SDR flowable composite.

## 4. Discussion

Argon lasers represent the first commercial source of coherent light which have been investigated for curing dental composites. The high energy emitted by these lasers at 488 nm wavelength is suitable for the CQ excitation. Many research efforts have been made over more than three decades, prompted by the independency of the quality of polymerisation on the tip-to-tooth distance, which represents the main future of a coherent and collimated beam and the main drawback of conventional quartz and LED LCUs. With regard to clinical devices, argon lasers have not been fully considered for several reasons, even if promising results were obtained. The cost of this laser is more than tenfold higher than conventional LCUs, and the equipment is bulky and heavy. Even though laser requires a shorter exposure time to cure composites [19], this feature is negatively balanced by the prolonged time required for the equipment to be ready before lighting [3]. Solid-state lasers have been recently developed based on GaN and InGaN [21], and, similarly to LED units, they are inexpensive with low electric power consumption. Compared with argon lasers, diode lasers are compact and lightweight [18].

From a technical point of view, the efficiency of an LCU can be defined by the ratio between the optical power output and the electric power. Hence, from Table 2, it can be easily computed that LED units have an efficiency between 6% and 10%, while the diode laser has an efficiency of 5%. Therefore, this type of laser is not so much less efficient than LEDs, it operates at low voltage, and a wireless compact device can be easily conceived. Of course, compared with conventional LCUs, the independence of the distance between the laser diode and the composite was also evaluated (Figure 2) [3]. 

Many efforts have been made to analyse the curing performance of different LCUs [49,50,51]. It is well known that lasers represent a valid alternative to LEDs and quartz units [3,18,20], but results are conflicting. Many literature studies support the hypothesis that similar or better performances are obtained with lasers [3,15,20], but some inconsistency has also been reported [22,24]. As composite light curing is conceived, light intensity and exposure time are the main variables to be considered, and the product between these two variables provides the light energy dose per unit area. It has been reported that similar composite properties are obtained using similar energy doses [52], although some differences may be found as high light intensity and short exposure time are used [12,26]. When using a laser unit, the main difficulty is the determination of the light intensity. In fact, this light source provides a very high radiance over a little spot, whereas the total optical power is known or can be easily measured (Table 2), the precise evaluation of the spot area is difficult, thus, the light intensity can be only roughly estimated [18,21]. Moreover, when defocusing the laser beam over a wider area, the light intensity distribution is not uniform (Figure 3a), and the evaluation of a mean intensity by means of the ratio between the optical power output and the irradiating area may provide underestimated results. To overcome this issue, a laser beam profile was considered (Figure 3). In particular, when using the laser spot for Mode I polymerisation, an almost elliptical projection having axis dimensions of 2.5 mm and 1 mm can be observed (Figure 3a). We used this beam oriented with the longer axis perpendicular to the longitudinal direction of the bending specimen. Thus, the width of the specimen was irradiated using an intensity between 90 mW/mm^2^ and 120 mW/mm^2^, and the whole specimen was polymerised by a continuous relative movement between the light source and the specimen (Mode I). A similar approach was used for the commercial LED unit in order to prevent the lack of homogeneity and the formation of several interfaces due to a “step-polymerisation”. Equation (3) was used to compute the energy dose provided to the specimen. This equation takes into account the engagement and the disengagement of the light beam with the specimen at the beginning and at the end of the Mode I curing process, respectively. The full engagement occurs at the time point t_1_, and at a given relative speed, this point depends on the beam diameter (about 1 mm and 8 mm for the laser unit and LED unit, respectively). Using Mode I curing process and Equation (3), it was possible to finely calibrate the energy dose provided by the different LCUs to the specimen.

The quality of the Mode I photopolymerisation process was assessed by the temperature profiles in the specimen (Figure 4 and Figure 5). For the diode laser process, a spot region at higher temperature level along the whole length of the specimen was observed (Figure 4). Instead, in the case of LED blue Mode I at higher temperature level, a much bigger region, along the whole length of the specimen, was found (Figure 5). In both cases, a uniform polymerisation was expected, as the temperature of every region of the specimen gradually reached a maximum value, and then gradually decreased. In particular, the gradual temperature decrease is represented by the remarked tail of any spot in Figure 4. This temperature decrease was related to the lower laser light intensity at the boundary of the beam (Figure 3a) which gradually cooled every point of the polymerised specimen heated during polymerisation. Therefore, the gradual temperature increase before polymerisation occurs, as well as the gradual temperature decrease after polymerisation simulated a soft start [53] and a soft light energy release [54], respectively. This feature of Mode I polymerisation should prevent thermal shrinkage stress, which may negatively affect the mechanical properties of specimens cured at high light intensity [26].

It is worth noting that temperature rise (Table 3) during laser Mode I curing was higher (*p* < 0.05) than that recorded for blue LED Mode I, while no difference was observed between temperature rise according to laser Mode II or LED Mode II polymerisation (Table 3). Of course, the higher values of temperature rise recorded for laser Mode I polymerisation may be addressed to the high light intensity (Figure 3a). Instead, using Mode II similar light intensity values from laser (Figure 3b) and LED were released to the specimen, and the similar values of temperature rise using laser Mode II or LED Mode II (Table 3) suggested the dependence of the temperature rise on the light intensity values, rather than on the curing source. 

Clearly, the intensity of the LED or laser irradiation is not the only factor influencing the temperature change. It is worth remembering that generally, the curing of composite resins produces a temperature rise as a consequence of the exothermic reaction, which contributes to the dynamic temperature profiles [13,14,26].

The inconsistency of our findings with literature results supporting lower temperature rise for lasers [16,18] may be ascribed to the estimation of light intensity. This parameter was computed as the ratio between the laser power output and the projected area for the defocused beam. Figure 3a clearly shows a non-uniform distribution of light intensity, and the ratio of the power output and the area of the projected area for the defocused beam leads to an underestimation of the light intensity value effectively involved in the curing process. Anyway, further research will be carried out to assess whether lasers, used in a pulsed modality, can provide significantly lower temperature increase during the polymerisation process.

Bending modulus and strength of EPH (Table 4) were consistent with previous findings [14], while the strength values obtained for SDR were slightly lower than those previously reported [31]. No statistically significant difference was observed between laser and LED polymerisation, thus suggesting that, at least for thin specimens, the quality of the polymerisation depends on the energy (dose) provided to the specimen. This result is consistent with another finding [22] supporting the hypothesis that the quality of laser-cured composites is similar to that of conventional curing. If the energy dose is higher than a minimum threshold, polymerisation occurring during the dark reaction phase increases mechanical properties, also reaching the values recorded for composites cured with high energy dose [14]. Further investigation will be carried out to determine differences among the properties of composites cured with laser and LED, in the very early stages after the photopolymerisation process.

On the other hand, statistically significant differences were observed among the mechanical properties of the different materials. In particular, the elastic modulus of EPH (Table 4) was significantly higher (*p* < 0.05) than DEI, CLM, and SDR composites. Also, the bending strength (Table 4) of EPH was higher (*p* < 0.05) than DEI and CLM. No difference was detected between the bending strength of EPH and SDR cured using Mode I process. However, the presence of Bis-GMA in the organic matrix and the greater amount of inorganic fillers make EPH stiffer than SDR, which also provided higher values of strain at break (*p* < 0.05) in comparison with all the other composites (Table 4), thus suggesting a higher toughness.

## 5. Conclusions

Based on the obtained results, the following conclusions were drawn:

1. Diode laser would be suitable for curing dental composites. Similarly to argon laser, diode laser photopolymerisation does not suffer from the tip-to-tooth distance. This feature is important for the polymerisation of composites deeply positioned into the tooth cavity.
2. By providing similar light energy dose, a significant difference in temperature rise was found for laser emitting intensity, that was higher than LED (Mode I).
3. By providing similar light energy dose, no significant difference in temperature rise was observed when the two light sources provided similar intensity (Mode II).
4. After 7 days since curing, bending properties of composites cured with laser and LED were similar.

## Figures and Tables

**Table 1:** Specifics and composition of the analysed restorative composites. The amount of particles is expressed as volume percentage.

Composite	Code	Type	Manufacturer	Organic Matrix	Inorganic Phase
Enamel plus Hri	EPH	UD2	Micerium	UDMA, Bis-GMA, BDDMA	Glass filler, Silicon dioxide (53 vol %)
Clearfil Majesty Esthetic	CLM	A2	Kuraray	Bis-GMA, Hydrophobic aromatic dimethacrylate	Silanated barium glass filler (66 vol %)
DEI	DEI	Enamel	DEI Italia	Bis-GMA, UDMA TEGDMA, EBPADMA	Vitreous fillers; pyrogenic silica
Smart dentine Replacement	SDR	Flowable Base	Dentsply	Modified UDMA, EBPADMA, TEGDMA	Barium and strontium alumino-fluoro-silicate glasses (45 vol %)

**Table 2:** Light sources used for curing restorative materials and measured characteristics: wavelength, optical power, electric voltage, electric current, electric power, optical energy.

Source	Manufacturer	Measured Wavelength (nm)	Optical Power (mW)	Electric Voltage (V)	Electric Current (A)	Electric Power (W)	Optical Energy (J)
Blue laser BM-200MW	Sothiclasers	451	185	12	0.31	3.72	4.85 (Mode I)
							4.93 (Mode II)
Blue LED Dental Starlight	Mectron	460	497	19	0.42	7.98	4.89 (Mode I)
Blue LED Light engine	458	Enfis Uno	4680	12	3.57	42.84	4.86 (Mode II)

**Table 3:** Temperature rise (°C) recorded during the photopolymerisation process for the analysed materials, according to light source and curing mode. All the results are reported in terms of mean values and standard deviation (in brackets).

Light Source	EPH	DEI	CLM	SDR
Laser (Mode I)	19.2 (±1.7)	15.4 (±1.1)	14.4 (±1.3)	18.7 (±1.5)
Laser (Mode II)	14.5 (±0.9)	10.1 (±0.7)	9.8 (±0.8)	14.6 (±1.2)
LED Mectron (Mode I)	16.3 (±1.3)	12.2 (±1.3)	12.1 (±1.4)	15.3 (±1.5)
LED Enfis (Mode II)	15.2 (±1.5)	9.5 (±0.8)	10.4 (±1.3)	16.2 (±1.4)

**Table 4:** Mechanical properties of restorative composites after 7 days since curing, according to light source and curing mode. All the results are reported in terms of mean values and standard deviation (in brackets).

	EPH			DEI			CLM			SDR		
LCU	Elastic Modulus (MPa)	Max. Stress (MPa)	Max. Strain (%)	Elastic Modulus (MPa)	Max. Stress (MPa)	Max. Strain (%)	Elastic Modulus (MPa)	Max. Stress (MPa)	Max. Strain (%)	Elastic Modulus (MPa)	Max. Stress (MPa)	Max. Strain (%)
Laser (Mode I)	8.6 (±0.7)	120.3 (±6.3)	2.2 (±0.1)	3.6 (±0.2)	61.9 (±4.1)	2.8 (±0.2)	6.0 (±0.7)	76.0 (±5.4)	1.4 (±0.1)	5.7 (±0.5)	107.5 (±5.9)	4.1 (±0.2)
Laser (Mode II)	8.4 (±0.8)	119.8 (±5.9)	2.1 (±0.2)	3.5 (±0.3)	60.2 (±4.8)	2.7 (±0.2)	5.9 (±0.5)	74.6 (±4.6)	1.4 (±0.1)	5.4 (±0.7)	105.8 (±6.1)	4.3 (±0.3)
LED Mectron (Mode I)	8.7 (±0.7)	121.9 (±5.8)	2.3 (±0.1)	3.8 (±0.2)	63.4 (±3.8)	2.9 (±0.1)	6.4 (±0.4)	77.8 (±4.3)	1.4 (±0.1)	5.7 (±0.4)	109.2 (±4.9)	4.4 (±0.2)
LED Enfis (Mode II)	8.1 (±0.7)	118.3 (±5.4)	2.2 (±0.1)	3.5 (±0.2)	56.7 (±5.3)	3.0 (±0.2)	6.3 (±0.5)	73.7 (±5.1)	1.3 (±0.1)	4.9 (±0.4)	93.0 (±6.8)	5.0 (±0.3)
